# Extracellular volume fraction mapping at 3T with non-rigid image co-registration

**DOI:** 10.1186/1532-429X-18-S1-P32

**Published:** 2016-01-27

**Authors:** Thu Thao Le, Shuo Zhang, Sven Kabus, Bao Ru Leong, Yiying Han, Derek J Hausenloy, Stuart Cook, Ru San Tan, Calvin W Chin

**Affiliations:** 1 grid.419385.20000000406209905National Heart Centre, Singapore, Singapore; 2 Philips Healthcare, Singapore, Singapore; 3 Philips Research, Hamburg, Germany; 4 grid.83440.3b0000000121901201University College London, London, UK; 5 grid.7445.20000000121138111Imperial College London, London, UK

## Background

MOLLI-based T1-mapping is a robust method for myocardial tissue characterization. Extracellular volume fraction (ECV), a marker of diffuse fibrosis, requires pre- and post-contrast T1 values. However, ECV mapping is not widely available because of complexities in motion correction and image co-registration. Here, we demonstrated ECV mapping using a novel approach for motion compensation at 3T.

## Methods

All patients underwent cardiovascular magnetic resonance (CMR) on a 3T system (Philips Ingenia). T1 maps were acquired in the basal and mid-cavity short-axis level, pre- and 20-min post-contrast (Gadovist 0.1 mmol/kg) with a 5s(3s)3s and 4s(1s)3s(1s)2s MOLLI acquisition scheme, respectively. A modified non-rigid, non-parametric registration method consisting of elastic registration steps was applied to generate motion-corrected T1 maps and subsequent ECV maps [1]. T1 error maps and overlay images were used as an indication for quality control. Global ECV values were expressed as mean+/-standard deviation (SD).

## Results

**Figure 1:**
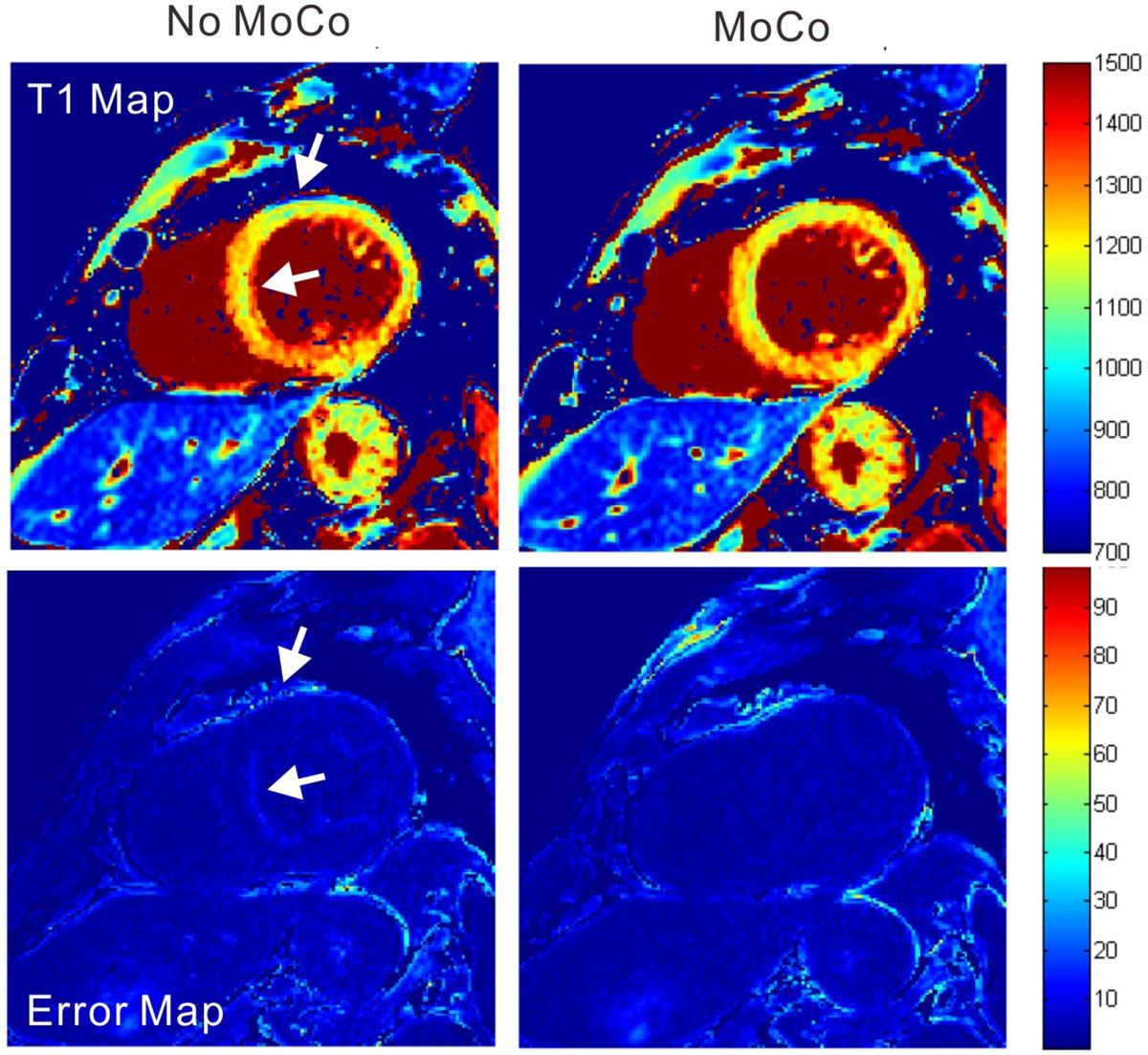
**Native myocardial T1 map without (left) and with (right) motion correction at 3T**. Arrows indicate motion misalignment that was corrected by the proposed approach.

**Figure 2:**
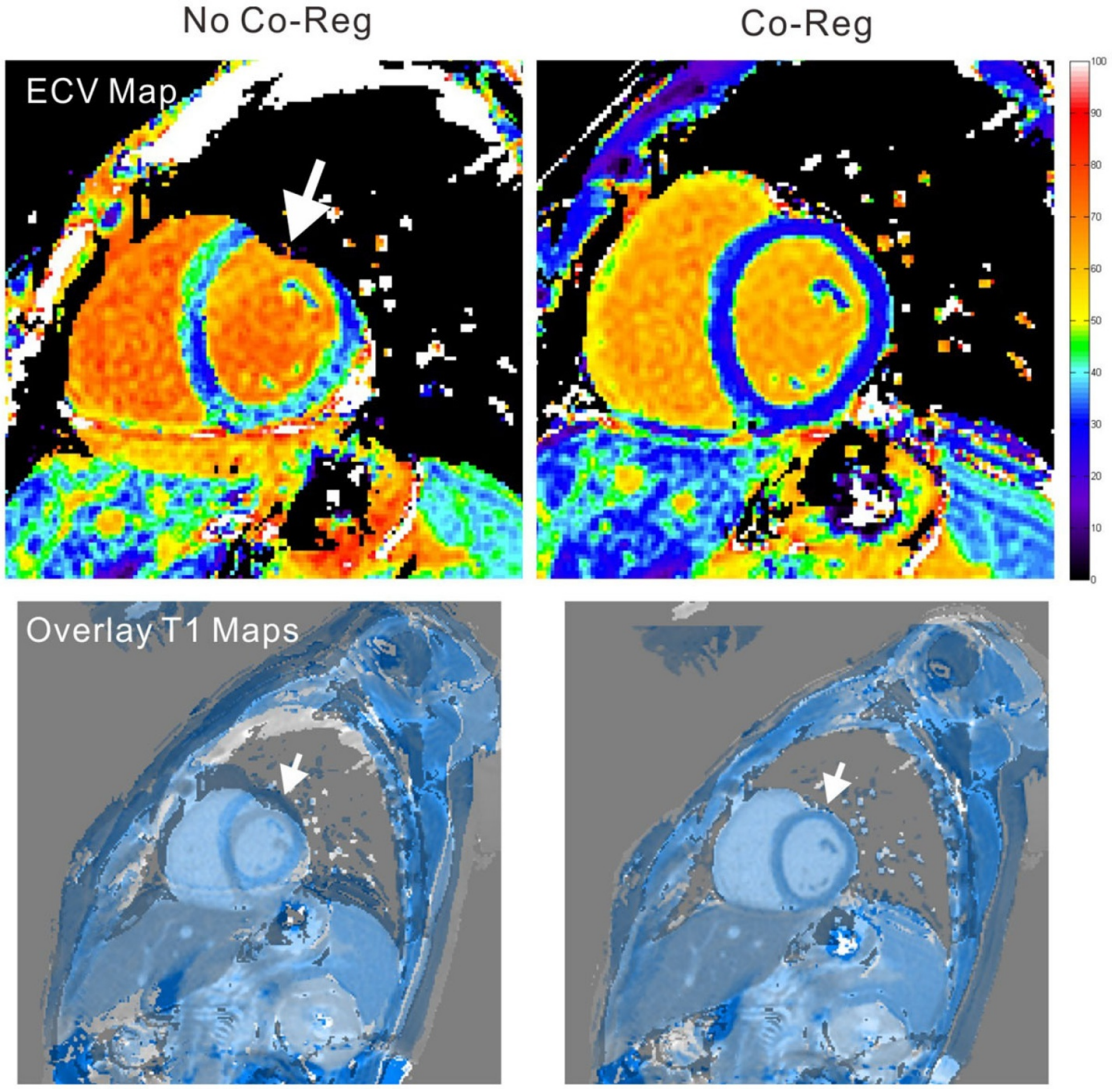
**Myocardial extracellular volume fraction (ECV) map without (left) and with (right) co-registration**. Arrows indicate motion misalignment that was corrected by the proposed approach.

A total of 33 ECV maps were obtained in 18 patients (mean age 47+/-19 years, 12 males): 10 with chronic myocardial infarction and 8 with dilated and hypertrophic cardiomyopathies. 28 cases (85%) demonstrated clear improvement in image quality after motion correction and co-registration. Two examples are shown in Figures 1 and 2. Global ECV values in the patients with myocardial infarction and cardiomyopathies were 34.9+/-4.9% and 36.4+/-6.0%, respectively.

## Conclusions

Automated motion correction and co-registration improved the quality of T1 and ECV maps at 3T, making ECV mapping feasible for clinical application.
